# The Effects of NaI, KBr, and KI Salts on the Vapor-Liquid Equilibrium of the H_2_O+CH_3_OH System

**DOI:** 10.3389/fchem.2020.00192

**Published:** 2020-04-07

**Authors:** Xianzhen Xu, Na Zhang, Yu Zhou, Yan Wang, Zonghua Wang

**Affiliations:** ^1^Shandong Sino-Japanese Center for Collaborative Research of Carbon Nanomaterials, College of Chemistry and Chemical Engineering, Qingdao University, Qingdao, China; ^2^Department of Avionics Engineering, Navel Aeronautical University Qingdao Branch, Qingdao, China

**Keywords:** water-methanol-salt, electrolyte solution, vapor–liquid equilibrium (VLE), thermodynamic, modeling

## Abstract

The vapor–liquid equilibrium (VLE) in chemical engineering is indispensable for the design of equilibrium separation processes such as distillation, absorption, extraction, and crystallization. VLE data were measured for H_2_O+CH_3_OH+NaI, H_2_O+CH_3_OH+KBr, and H_2_O+CH_3_OH+KI systems. By analyzing and summarizing the results of H_2_O+Methanol+Alkali metal halide systems, the salt effects of NaI, KBr, and KI on the vapor–liquid equilibrium were obtained. Simultaneously, a model based on the NRTL equation (non-random two liquid) was proposed to correlate and calculate the VLE for the systems. In addition, the assumption of solvation based on hydration was introduced in this model. The proposed model can be successfully used to calculate the VLE for H_2_O+Methanol+Alkali metal halide systems.

## Introduction

Vapor–liquid equilibrium (VLE), solid–liquid equilibrium (SLE), and liquid–liquid equilibrium (LLE) are important in industry, natural processes, chemistry, and other fields. The VLE for electrolyte systems and, more specifically, for mixed solvent electrolyte mixtures (such methanol-water-salt systems) are of considerable importance to a variety of fields, such as the extractive distillation of salt-containing liquids (Iliuta et al., 2000). There has been an increase in the amount of research into the phase equilibrium of electrolyte and non-electrolyte solutions.

Phase equilibrium and the thermodynamics of electrolyte solutions have been studied for decades, including activity coefficient, phase equilibrium data, and activity coefficient models. The Wilson model (Aebischer et al., 2018), NRTL model (Farajnezhad et al., 2016), and UNIQUAC model (Pereira et al., 2019) can be used to accurately calculate thermodynamic properties of non-electrolyte solutions. The Lu–Maurer model (Qian et al., 2011; Kontogeorgis et al., 2018), homsen's model (Pitzer, 2018), Pitzer's model (Hossain et al., 2016), ElecNRTL model (Puentes et al., 2018; Das et al., 2019), OLI model (Xu et al., 2016), and Xu's model (Yuan et al., 2019) have been successfully used to calculate the thermodynamic properties and the phase equilibrium for electrolyte solutions. In recent years, many researchers have begun to study the VLE of mixed-solvent electrolytes, and the VLE is important in the design of separation processes. Yang and Lee (1998) studied the VLE of H_2_O+CH_3_OH+NaCl, H_2_O+CH_3_OH+NaBr, and H_2_O+CH_3_OH+KCl through an experiment. The LIQUAC model (Li et al., 2010; Mohs and Gmehling, 2013) has been proposed to calculate the phase equilibria of mixed-solvent electrolyte solutions. In this model, Yan et al. treated the solutes as non-electrolyte solution interactions. Zhong et al. (2017) combined the UNIFAC model with the LIQUAC model and then developed the LIFAC model. Chen and Song (2004) proposed a modified model based the electrolyte NRTL model; it can be used to calculate the ionic activity coefficients of mixed-solvent electrolyte systems. These studies reported some experimental data and modified models. Experimental data were relatively abundant for single or mixed electrolyte aqueous systems (Yang and Lee, 1998), but the phase equilibrium data of the methanol-water-salt system with a wide range of pressures and temperatures were still rare. Such systems may be of practical importance or of interest to the development of a general electrolyte solution model. The models combine local composition activity coefficient models with either Debye-Hückel's law or the modifications of Debye-Hückel's law. Researchers have expanded the range of applications. The models can be used to calculate binary, multi-component electrolyte solutions at high temperatures and high concentrations. In general, there are great challenges in the research of mixed-solvent electrolytes, such as unavailable experimental data, unobtained salt–salt interaction parameters, and limited predictive capability.

In this work, we measured the VLE data of H_2_O+CH_3_OH+NaI, H_2_O+CH_3_OH+KBr, and H_2_O+CH_3_OH+KI systems. Then, a modified model was proposed to correlate the VLE of mixed solvent electrolyte systems.

## Experimental Section

### Materials

The NaI (AR, 99.5%), KBr (AR, 99.5%), KI (AR, 99%), and CH_3_OH (AR, 99.5%) of the solute are anhydrous, and they were purchased from Sinopharm Chemical Reagent Co. Ltd, Shanghai, China. Distilled water (18.2 Ω cm) was used for the preparation of solutions.

### Apparatus and Procedures

**Figure 1:**
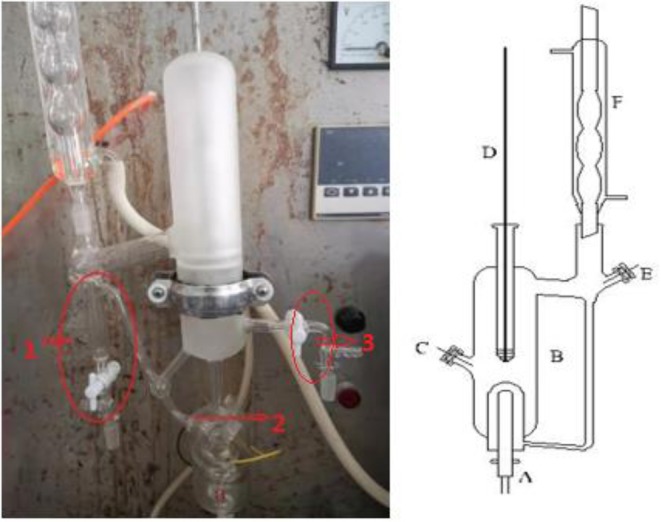
Schematic diagram of the VLE apparatus used in this work: (1) vapor sampling port, (2) Sample level, (3) liquid sampling port, (A) heating mantle, (B) equilibrium still, (C) sampling port, (D) thermometer well, (E) sampling port, (F) condenser, REPRODUCED from the Wang et al. (2019) under the Creative Commons CC-BY license.

We used a circulation glass ebulliometer to measure the VLE, and the capacity of the ebulliometer was 40 cm^3^, as shown in Figure 1 (Wang et al., 2019). The main experimental instruments included a vacuum pump in the ebulliometer (40 cm^3^, Tianjin Wuqing Beiyang Chemical Factory), a pressure controller (Ruska Series 7000, Ruska Instrument Corp., Houston), a heating mantle, and a temperature controller (Model SRS13A, SHIMADEN, Japan).

During the experiments, the sample was placed into the glass ebulliometer, and the sample was added to the height of mark 2, as shown in Figure 1. The sample was then heated by the heating mantle controlled by the temperature controller. The operation pressure was controlled by the vacuum pump, the pressure sensor, and control valve. The vapor sample was condensed in a spherical condenser (length 40 cm) and then returned to the mixing chamber for recirculation. The time was 0.5–1 h in the first equilibrium, and the following equilibrium time was 10–20 min. The judging standard of the VLE is an important factor. The condensate reflux of the ebulliometer was controlled at 2–3 drops per second and was stably refluxed for approximately 15 min to establish an equilibrium state. After the VLE was reached, we recorded the temperature and pressure. At last, the component results of the vapor sample were tested through the gas chromatography with a TCD detector and a FFAP capillary chromatogram column.

The reliability of measurement has been verified in literature (Xu et al., 2018, 2019) (i.e., H_2_O+CaCl_2_ and H_2_O+C_2_H_5_OH). The experimental VLE data for three ternary systems (i.e., H_2_O+CH_3_OH+NaI, H_2_O+CH_3_OH+KBr, and H_2_O+CH_3_OH+KI) were listed in Tables 1–3. In the tables, x and y are the components in the liquid phase and in the vapor phase, respectively.

**Table 1:** Experimental VLE data for the H_2_O(1)+CH_3_OH(2)+NaI(3) system.

**T/K**	**P/kPa**	**x_**1**_**	**x_**2**_**	**x_**3**_**	**y_**1**_**
314.45	15.47	0.78	0.20	0.02	0.357
314.25	15.77	0.75	0.20	0.05	0.338
314.40	16.23	0.72	0.20	0.08	0.320
313.65	15.63	0.71	0.20	0.09	0.317
313.95	16.09	0.68	0.20	0.12	0.309
311.60	14.38	0.64	0.20	0.16	0.306
317.15	13.82	0.88	0.10	0.02	0.576
316.75	13.63	0.82	0.10	0.08	0.557
316.15	13.40	0.76	0.10	0.14	0.528
316.05	13.45	0.69	0.10	0.21	0.470
315.85	13.29	0.65	0.10	0.25	0.477
315.95	13.35	0.63	0.10	0.27	0.470
314.75	22.45	0.53	0.45	0.02	0.216
315.00	23.03	0.52	0.45	0.03	0.187
316.15	24.60	0.51	0.45	0.04	0.187
315.45	24.03	0.50	0.45	0.05	0.187
315.15	24.21	0.48	0.45	0.07	0.182
315.25	24.58	0.47	0.45	0.08	0.168
321.80	27.14	0.67	0.31	0.02	0.287
319.95	25.50	0.64	0.31	0.05	0.267
322.15	29.15	0.61	0.31	0.08	0.249
320.75	27.46	0.60	0.31	0.09	0.251
323.15	31.43	0.57	0.31	0.12	0.227
320.15	27.79	0.53	0.31	0.16	0.218
328.70	22.99	0.90	0.08	0.02	0.565
328.20	22.44	0.83	0.08	0.09	0.567
330.75	25.70	0.78	0.08	0.14	0.547
330.25	25.36	0.74	0.08	0.18	0.495
331.65	27.33	0.67	0.08	0.25	0.477
332.80	28.92	0.65	0.08	0.27	0.465
336.10	45.72	0.76	0.22	0.02	0.388
335.45	45.22	0.73	0.22	0.05	0.355
335.40	45.94	0.70	0.22	0.08	0.347
335.15	45.54	0.69	0.22	0.09	0.341
334.65	45.07	0.66	0.22	0.12	0.328
334.45	45.22	0.62	0.22	0.16	0.319
353.75	55.47	0.95	0.03	0.02	0.806
353.35	53.53	0.88	0.03	0.09	0.777
352.90	53.44	0.82	0.03	0.15	0.737
354.50	56.98	0.8	0.03	0.17	0.727
354.95	57.64	0.76	0.03	0.21	0.705
356.55	58.81	0.65	0.03	0.32	0.655
341.50	76.15	0.51	0.47	0.02	0.224
341.15	75.55	0.5	0.47	0.03	0.215
341.05	75.98	0.49	0.47	0.04	0.217
340.40	74.91	0.48	0.47	0.05	0.207
341.15	77.84	0.47	0.47	0.06	0.205
341.95	81.16	0.46	0.47	0.07	0.206
352.45	101.31	0.47	0.47	0.06	0.337

*Standard uncertainties u were u(P) = 0.01 kPa, u(T) = 0.05 K, and u(y) = 0.01%, respectively*.

*$\text{u}\left(x_{1}\right)=\frac{u\left(m_{1}\right)/18.0152}{m_{1}/18.0152+m_{2}/32.04186+m_{3}/149.89}$*.

*$\text{u}\left(x_{2}\right)=\frac{u\left(m_{2}\right)/32.04186}{m_{1}/18.0152+m_{2}/32.04186+m_{3}/149.89}$*.

*$\text{u}\left(x_{3}\right)=\frac{u\left(m_{3}\right)/149.89}{m_{1}/18.0152+m_{2}/32.04186+m_{3}/149.89}$*.

**Table 2:** Experimental VLE data of the H_2_O(1)+CH_3_OH(2)+KBr(3) system.

**T/K**	**P/kPa**	**x_**1**_**	**x_**2**_**	**x_**3**_**	**y_**1**_**
314.25	17.27	0.78	0.22	0.00	0.367
314.05	17.48	0.74	0.22	0.04	0.346
314.25	18.01	0.71	0.22	0.07	0.332
313.45	17.38	0.69	0.22	0.09	0.327
313.75	17.91	0.68	0.22	0.10	0.315
311.45	15.75	0.64	0.21	0.15	0.307
316.95	13.75	0.92	0.08	0.00	0.591
316.55	13.41	0.89	0.08	0.03	0.583
315.95	13.07	0.86	0.08	0.06	0.567
315.85	13.65	0.82	0.09	0.09	0.528
315.65	13.07	0.79	0.08	0.13	0.536
315.75	13.20	0.74	0.08	0.18	0.513
314.55	24.10	0.53	0.47	0.00	0.210
314.85	24.69	0.52	0.47	0.01	0.202
315.95	26.33	0.51	0.47	0.02	0.197
315.25	25.49	0.51	0.46	0.03	0.195
314.95	25.67	0.49	0.46	0.05	0.185
315.05	26.06	0.48	0.46	0.06	0.180
321.65	28.76	0.68	0.32	0.00	0.300
319.75	27.08	0.65	0.32	0.03	0.275
321.95	30.87	0.62	0.32	0.06	0.262
320.55	29.13	0.61	0.32	0.07	0.255
322.95	32.82	0.60	0.32	0.08	0.253
319.95	28.48	0.59	0.31	0.10	0.248
328.55	25.36	0.91	0.09	0.00	0.578
328.05	23.74	0.89	0.08	0.03	0.593
330.55	26.89	0.84	0.08	0.08	0.576
330.05	27.45	0.81	0.09	0.1	0.539
331.45	28.43	0.79	0.08	0.13	0.55
332.65	30.38	0.73	0.08	0.19	0.518
335.95	47.63	0.78	0.22	0.00	0.396
335.25	47.12	0.75	0.22	0.03	0.373
335.25	47.85	0.72	0.22	0.06	0.358
334.95	47.45	0.71	0.22	0.07	0.353
334.45	46.96	0.68	0.22	0.1	0.339
334.25	46.16	0.65	0.21	0.14	0.333
353.55	58.03	0.97	0.03	0.00	0.808
353.15	56.01	0.90	0.03	0.07	0.789
352.75	55.91	0.84	0.03	0.13	0.750
354.35	59.5	0.82	0.03	0.15	0.741
354.75	60.17	0.78	0.03	0.19	0.719
356.35	63.45	0.75	0.03	0.22	0.707
341.35	78.60	0.53	0.47	0.00	0.240
340.95	78.00	0.52	0.47	0.01	0.231
340.85	77.77	0.52	0.46	0.02	0.228
340.25	76.69	0.51	0.46	0.03	0.221
340.95	79.65	0.50	0.46	0.04	0.215
341.75	83.00	0.49	0.46	0.05	0.210
350.85	101.32	0.68	0.25	0.07	0.340

*Standard uncertainties u were u(P) = 0.01 kPa, u(T) = 0.05 K, and u (y) = 0.01%, respectively*.

*$\text{u}\left(x_{1}\right)=\frac{u\left(m_{1}\right)/18.0152}{m_{1}/18.0152+m_{2}/32.04186+m_{3}/149.89}$*.

*$\text{u}\left(x_{2}\right)=\frac{u\left(m_{2}\right)/32.04186}{m_{1}/18.0152+m_{2}/32.04186+m_{3}/149.89}$*.

*$\text{u}\left(x_{3}\right)=\frac{u\left(m_{3}\right)/149.89}{m_{1}/18.0152+m_{2}/32.04186+m_{3}/149.89}$*.

**Table 3:** Experimental VLE data of the H_2_O(1)+CH_3_OH(2)+KI(3) system.

**T/K**	**P/kPa**	**x_**1**_**	**x_**2**_**	**x_**3**_**	**y_**1**_**
315.05	15.87	0.78	0.22	0.00	0.362
314.85	16.17	0.78	0.21	0.01	0.341
315.00	16.63	0.77	0.21	0.02	0.327
314.25	16.03	0.69	0.21	0.10	0.322
314.55	16.49	0.68	0.21	0.11	0.310
312.20	14.78	0.64	0.21	0.15	0.302
317.75	14.22	0.89	0.08	0.03	0.586
317.35	14.03	0.89	0.08	0.03	0.578
316.75	13.8	0.86	0.08	0.06	0.562
316.65	13.85	0.82	0.09	0.09	0.523
316.45	13.69	0.79	0.08	0.13	0.531
316.55	13.75	0.74	0.08	0.18	0.508
315.35	22.85	0.53	0.47	0.00	0.205
315.60	23.43	0.52	0.47	0.01	0.197
316.75	25.00	0.51	0.47	0.02	0.192
316.05	24.43	0.51	0.46	0.03	0.190
315.75	24.61	0.49	0.46	0.05	0.180
315.85	24.98	0.48	0.46	0.06	0.175
322.40	27.54	0.68	0.32	0.00	0.295
320.55	25.90	0.65	0.32	0.03	0.270
322.75	29.55	0.62	0.32	0.06	0.257
321.35	27.86	0.61	0.32	0.07	0.250
323.75	31.83	0.60	0.32	0.08	0.248
320.75	28.19	0.59	0.31	0.10	0.243
329.30	23.39	0.91	0.09	0.00	0.573
328.80	22.84	0.89	0.08	0.03	0.588
331.35	26.10	0.84	0.08	0.08	0.571
330.85	25.76	0.81	0.09	0.10	0.534
332.25	27.73	0.79	0.08	0.13	0.545
333.40	29.32	0.73	0.08	0.19	0.513
336.70	46.12	0.78	0.22	0.00	0.391
336.05	45.62	0.75	0.22	0.03	0.368
336.00	46.34	0.72	0.22	0.06	0.353
335.75	45.94	0.71	0.22	0.07	0.348
335.25	45.47	0.68	0.22	0.10	0.334
335.05	45.62	0.65	0.21	0.14	0.328
354.35	55.87	0.97	0.03	0.00	0.803
353.95	53.93	0.90	0.03	0.07	0.784
353.50	53.84	0.84	0.03	0.13	0.745
355.10	57.38	0.82	0.03	0.15	0.736
355.55	58.04	0.78	0.03	0.19	0.714
357.15	59.21	0.75	0.03	0.22	0.702
342.10	76.55	0.53	0.47	0.00	0.235
341.75	75.95	0.52	0.47	0.01	0.226
341.65	76.38	0.52	0.46	0.02	0.223
341.00	75.31	0.51	0.46	0.03	0.216
341.75	78.24	0.50	0.46	0.04	0.210
342.55	81.56	0.51	0.47	0.02	0.205
353.05	101.31	0.69	0.25	0.06	0.335

*Standard uncertainties u were u(P) = 0.01 kPa, u(T) = 0.05 K, and u(y) = 0.01%, respectively*.

*$\text{u}\left(x_{1}\right)=\frac{u\left(m_{1}\right)/18.0152}{m_{1}/18.0152+m_{2}/32.04186+m_{3}/149.89}$*.

*$\text{u}\left(x_{2}\right)=\frac{u\left(m_{2}\right)/32.04186}{m_{1}/18.0152+m_{2}/32.04186+m_{3}/149.89}$*.

*$\text{u}\left(x_{3}\right)=\frac{u\left(m_{3}\right)/149.89}{m_{1}/18.0152+{\text{m}}_{2}/32.04186+m_{3}/149.89}$*.

## Model Description

### Modification of Xu's Model for Mixed Solvent Electrolyte Systems

Xu's model (Yuan et al., 2019) can be employed to correlate and predict the VLE for electrolyte solution systems. In this work, a modified Xu's model was proposed to be used to calculate the VLE for mixed solvent electrolyte systems. The model for the excess Gibbs energy was expressed by the NRTL term. For mixed solvent electrolyte system, we added the solvent-salt terms and the solvent-solvent terms in the proposed model (Xu et al., 2016). Then, the activity coefficients were calculated by the excess Gibbs energy of the solvent-salt term and solvent-solvent term. For example, in a solvent 1-solvent 2-salt system

(1) $$\begin{array}{l} \frac{n_{t}G}{RT}=\;n_{1}n_{3}\left(\frac{\tau_{1,3}G_{1,3}}{n_{3}+n_{1}G_{1,3}}+\frac{\tau_{3,1}G_{3,1}}{n_{1}+n_{3}G_{3,1}}\right)\\ +n_{2}n_{3}\left(\frac{\tau_{2,3}G_{2,3}}{n_{3}+n_{2}G_{2,3}}+\frac{\tau_{3,2}G_{3,2}}{n_{2}+n_{3}G_{3,2}}\right)\;\\ +n_{1}n_{2}\left(\frac{\tau_{1,2}G_{1,2}}{n_{2}+n_{1}G_{1,2}}+\frac{\tau_{2,1}G_{2,1}}{n_{1}+n_{2}G_{2,1}}\right) \end{array}$$

(2) $$\begin{array}{l} G_{i,j}=\exp\left(-\alpha_{}\tau_{i,j}\right) \end{array}$$

This approach has been used to calculate activity coefficient between 298 and 355 K. For correlating data at different temperatures, a temperature dependence of the parameters τ_*i, j*_ and τ_*i, j*_ was used in which

(3) $$\begin{array}{l} \tau_{i,j}\;\text{=}\;\tau_{i,j}^{0}+\tau_{i,j}^{1}/T \end{array}$$

where subscript 1, 2, and 3 are solvent 1, solvent 2, and salt, respectively; *nt* is the molar of solute; and solvent *mx* is the total molality of solute, α = 0.3. The reference state of the activity coefficients in the excess Gibbs energy model is γi → 1 as *x*_*i*_ (=*n*_*i*_*/n*_*t*_) → 1.

In Equation 10, the solvation of solvent based on the hydration of Xu's model was introduced:

(4) $$\begin{array}{l} n_{1}=n_{1}^{0}-Z_{1}*n_{3}^{0} \end{array}$$

(5) $$\begin{array}{l} n_{2}=n_{2}^{0}-Z_{2}*n_{3}^{0} \end{array}$$

(6) $$\begin{array}{l} n_{3}=n_{3}^{0} \end{array}$$

where *n*_1_, *n*_2_, and *n*_3_ are active contents; $n_{1}^{0}$, $n_{2}^{0}$, and $n_{3}^{0}$ are actual contents; and *Z*_1_ and *Z*_2_ are solvation parameters.

The final equation can be deduced:

(7) $$\begin{array}{l} \ln\;\gamma_{1}=n_{3}^{2}\left(\tau_{3,1}{\left(\frac{G_{3,1}}{n_{1}+n_{3}G_{3,1}}\right)}^{2}+\tau_{1,3}{\left(\frac{G_{1,3}}{n_{3}+n_{1}G_{1,3}}\right)}^{2}\right)\\ \text{+}n_{2}^{2}\left(\tau_{2,1}{\left(\frac{G_{2,1}}{n_{1}+n_{2}G_{2,1}}\right)}^{2}+\tau_{1,2}{\left(\frac{G_{1,2}}{n_{2}+n_{1}G_{1,2}}\right)}^{2}\right)\; \end{array}$$

(8) $$\begin{array}{l} \ln\;\gamma_{2}=n_{3}^{2}\left(\tau_{3,2}{\left(\frac{G_{3,1}}{n_{1}+n_{3}G_{3,1}}\right)}^{2}+\tau_{2,3}{\left(\frac{G_{1,3}}{n_{3}+n_{1}G_{1,3}}\right)}^{2}\right)\\ \text{+}n_{1}^{2}\left(\tau_{1,2}{\left(\frac{G_{1,2}}{n_{2}+n_{1}G_{1,2}}\right)}^{2}+\tau_{2,1}{\left(\frac{G_{2,1}}{n_{1}+n_{2}G_{2,1}}\right)}^{2}\right) \end{array}$$

In the final model (Equations 7 and 8), parameters, $\tau_{2,1}^{0}$, $\tau_{3,1}^{0}$, $\tau_{2,3}^{0}$, $\tau_{3,2}^{0}$, $\tau_{2,1}^{1}$, $\tau_{3,1}^{1}$, $\tau_{2,3}^{1}$, $\tau_{3,2}^{1}$, *Z*_1_, and *Z*_2_ were fitted to the literature data, and the parameters can be used to calculate the activity coefficient for mixed solvent electrolyte systems between 298 and 355 K. Eight model parameters were used to fit the VLE data for one mixed electrolyte system at one temperature. The calculation software of this work was *1stopt 7.0* (7D-Soft High Technology Inc.), and the calculation algorithm was *Universal Global Algorithm*.

## Results and Discussion

**Figure 2:**
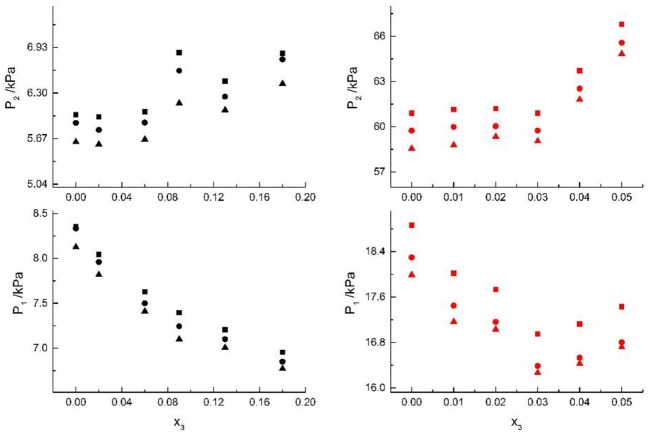
VLE of H2O (1)+CH3OH (2)+NaCl (3): ■ indicates the Literature data (Yang and Lee, 1998); H2O (1)+CH3OH (2)+NaBr (3): • indicates the Literature data (Xu et al., 2018); H2O(1)+CH3OH(2)+NaI(3): ▲. Filled symbols (Black: x2 = 0.08 and T = 316 K; Red: x2 = 0.46 and T = 341K) indicate the experimental data.

**Figure 3:**
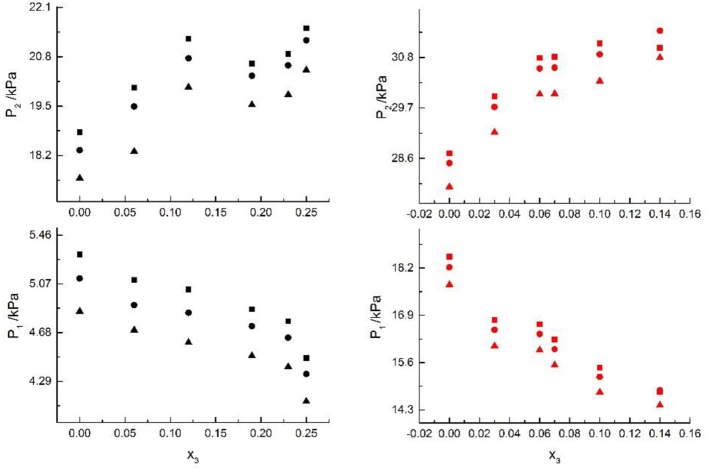
VLE of H2O(1)+ CH3OH(2)+KCl(3): ■ indicate the Literature data (Xu et al., 2018); H2O(1)+ CH3OH(2)+KBr(3): • indicate experimental data; H2O(1)+ CH3OH(2)+KI(3): ▲. Filled symbols (black: x2 = 0.45 and T = 315 K; red: x2 = 0.22 and T = 335 K) indicate experimental data.

The experimental data for three ternary systems (i.e., H_2_O+CH_3_OH+NaI, H_2_O+CH_3_OH+KBr, and H_2_O+CH_3_OH+KI) at different molality are listed in Tables 1–3. Meanwhile, we analyzed and summarized the results of H_2_O+CH_3_OH+NaCl (Yang and Lee, 1998), H_2_O+CH_3_OH+NaBr (Xu et al., 2018), H_2_O+CH_3_OH+NaI, H_2_O+CH_3_OH+KCl (Xu et al., 2018), H_2_O+CH_3_OH+KBr, and H_2_O+CH_3_OH+KI shown in Figures 2, 3, and we obtained the possible relationship between the solubility of salt and the VLE.

We then studied the thermodynamic model for mixed solvent electrolyte systems and proposed the modified NRTL model to correlate the VLE for the systems. Equations (7) and (8), the Yang's model (Yang and Lee, 1998), the Iliuta's model (Kumagae et al., 1992), the Kumagae's model (Robinson and Stokes, 2012), and Xu's model (Xu et al., 2018) were used to correlate VLE data in mixed-solvent electrolyte systems. Seven salts (i.e., NaCl, NaBr, NaI, KCl, KBr, KI, and CaCl2) and four solvents (i.e., water, methanol, ethanol, and 1-propanol) were chosen, and the VLE behaviors of 11 mixed-solvent electrolyte ternary systems were researched.

H_2_O+CH_3_OH+NaCl (Yang and Lee, 1998), H_2_O+CH_3_OH+NaBr (Xu et al., 2018), H_2_O+CH_3_OH+NaI, H_2_O+CH_3_OH+KCl (Xu et al., 2018), H_2_O+CH_3_OH+KBr, and H_2_O+CH_3_OH+KI systems were chosen to study the VLE of H_2_O+methanol+alkali metal halide systems, as shown in Figures 2, 3.

From the Tables and Figures, we can see that the VLE are similar in the alkali metal systems. For the H_2_O+CH_3_OH+NaCl, H_2_O+CH_3_OH+NaBr, and H_2_O+CH_3_OH+NaI systems, as the salt concentration x_3_ increased under the condition (x_2_ = 0.08 and T = 316 K), P_1_ of water decreased, and P_2_ of methanol rose regularly. As the salt concentration x_3_ increased under the condition (x_2_ = 0.46 and T = 341K), P_1_ of water decreased first and then rose, and P_2_ of methanol rose regularly. For the H_2_O+CH_3_OH+KCl, H_2_O+CH_3_OH+KBr, and H_2_O+CH_3_OH+KI systems, as the salt concentration x_3_ increased under the condition (x_2_ = 0.45 and T = 315K), P_1_ of water decreased, and P_2_ of methanol rose regularly. As the salt concentration x_3_ increased under the condition (x_2_ = 0.22 and T = 335K), P_1_ of water decreased, and P_2_ of methanol rose. Through the above analysis, we found that the solubility of salt is an important factor affecting the VLE.

### Results of the New Model

Parameters, $\tau_{2,1}^{0}$, $\tau_{3,1}^{0}$, $\tau_{2,3}^{0}$, $\tau_{3,2}^{0}$, $\tau_{2,1}^{1}$, $\tau_{3,1}^{1}$, $\tau_{2,3}^{1}$, $\tau_{3,2}^{1}$, *Z*_1_, and *Z*_2_were obtained from the correlation of the experimental and literature data, as listed in Table 4. The results of correlation for 11 mixed solvent electrolyte systems were listed in Table 5 in the form of mean deviation between literature and calculated value. It can be seen from Table 5 that *dY* ≤ 0.24 kPa, and the mean value of *dY* = 0.11 kPa; *dP* ≤ 3.79%, and the mean value of *dP* = 2.38%. *dY* and *dP* were calculated via equations:

(9) $$\begin{array}{l} dY=\left(1/N\right)\sum |P_{\exp}-P_{cal}| \end{array}$$

(10) $$\begin{array}{l} dP=\left(1N\right)\sum |P_{\exp}-P_{cal}|/P_{\exp}\times 100\% \end{array}$$

where *N* is the data point number, and *P*_*exp*_ and *P*_*cal*_ are experimental vapor pressure and calculated vapor pressure, respectively.

**Table 4:** Model parameters of some solvents-salt systems.

**Systems**	***$\tau_{1,2}^{0}$***	***$\tau_{2,1}^{0}$***	***$\tau_{1,3}^{0}$***	***$\tau_{3,1}^{0}$***	***$\tau_{2,3}^{0}$***	***$\tau_{3,2}^{0}$***	***$\tau_{1,2}^{1}$***	***$\tau_{2,1}^{1}$***	***$\tau_{1,3}^{1}$***	***$\tau_{3,1}^{1}$***	***$\tau_{2,3}^{1}$***	***$\tau_{3,2}^{1}$***	***Z_**1**_***	***Z_**2**_***
H_2_O+Methanol+NaCl	0.24	0.29	7.82	−3.71	3.69	0.38	227.5	−166.92	−1268.09	645.09	5349.32	−133.55	0	0.48
H_2_O+Methanol+NaBr	14.23	−8.13	−406.33	−18.03	23.05	−1.57	−4302.11	2540.2	151284.16	6548.14	−2235.55	600.81	0	0.186
H_2_O+Methanol+NaI	19.72	−9.12	−2.98	1.71	−7.43	−0.49	−6002.7	2788.76	1474.4	157.8	2976.7	−148.53	−3.02	0.39
H_2_O+Methanol+KCl	−0.15	−1255.78	12.21	−831.15	11.41	−139.46	233.17	448745.29	4047.21	298489.24	−2243.25	49032.8	0	−1.71
H_2_O+Methanol+KBr	3.01	−0.61	14.07	−42.26	−1.16	−0.23	−482.5	−10.87	−2838.77	14535.6	15.36	698.72	−8.31	0.17
H_2_O+Methanol+KI	18.06	−7.52	7	231.55	−707.4	−30.35	−5177	2118.6	−1129.2	−73112.7	243350.1	12396.3	−11	−0.34
H_2_O+Methanol+CaCl_2_	97.04	−29.58	49.65	79.21	−123.03	9.9	−26974.68	9203.11	−15365.74	−22079.96	26195.28	−2634.7	0.018	−0.077
H_2_O+Ethanol+CaCl_2_	−2.51	5.62	−25.15	−6.75	−38487.56	54.94	1314.8	−2134.15	10612.48	−493.9	726992.85	−15459.5	20	−0.12
Methanol+Ethanol+CaCl_2_	−104.8	−20.2	1.65	−77.92	12.14	−14.78	0.46	−0.11	−0.022	0.13	−0.21	−0.47	0.34	0.035
Methanol+1–propanol +CaCl_2_	−2.83	3.43	2.8	−0.0027	−485.52	−28.51	311.89	−112.32	−109.12	5.12	153053.58	1554.08	0.93	3948
Ethanol+1–propanol +CaCl_2_	3.16	869725.35	0.19	1020346	2.47	−29.56	50.25	−6350134.1	−3.27	−579512.5	41780.24	−134.48	0.84	−24836.53

**Table 5:** Correlation of VLE data for mixed-solvent systems at 298.15 K.

**Systems**	**Reference and experiment**	**Salt concentration**	**Data points**	**This work**	
				***dY*/kPa**	***dP*/%**
H_2_O+Methanol+NaCl	12,17 and 22	0–4 (mol/kg)	70	0.13	1.11
H_2_O+Methanol+NaBr	12,17 and 22	0–6 (mol/kg)	60	0.24	1.87
H_2_O+Methanol+NaI	Experiment	0–8 (mol/kg)	50	0.11	1.05
H_2_O+Methanol+KCl	12,17 and 22	0–2 (mol/kg)	70	0.15	1.22
H_2_O+Methanol+KBr	Experiment	0–4 (mol/kg)	50	0.09	0.92
H_2_O+Methanol+KI	Experiment	0–4 (mol/kg)	50	0.12	1.12
H_2_O+Methanol+CaCl_2_	18	0–15% (mass fraction)	40	0.14	3.79
H_2_O+Ethanol+CaCl_2_	18	0–15% (mass fraction)	20	0.05	2.32
Methanol+Ethanol+CaCl_2_	18	0–15% (mass fraction)	20	0.10	2.20
Methanol+1-propanol+CaCl_2_	18	0–15% (mass fraction)	36	0.06	3.42
Ethanol +1-propanol + CaCl_2_	18	0–15% (mass fraction)	36	0.04	3.14
Mean value				0.11	2.01

*dY = (1/N)∑|P_exp_-P_cal_|, where N is the number of data points*.

*dP = (1/N) ∑|P_exp_-P_cal_|/P_exp_ × 100%, where N is the number of data points*.

**Figure 4:**
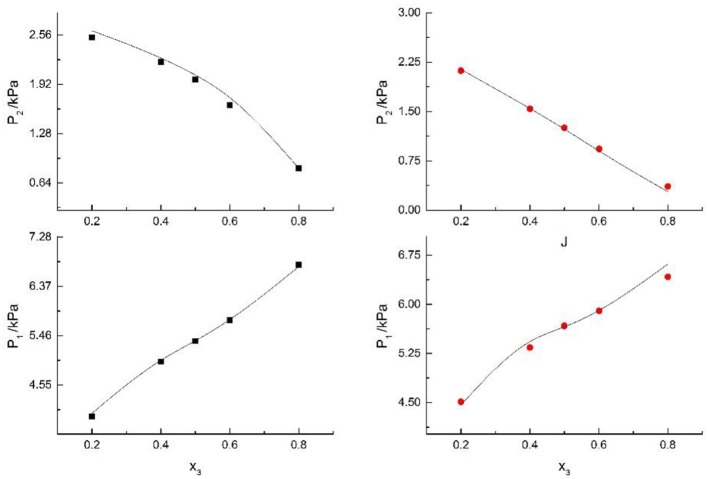
Correlation of VLE data of H2O(1)+ CH3OH(2)+ CaCl2(3) system. Filled symbols (■x2 = 0.05 and T = 298.15 K; •x2 = 0.15 and T = 298.15 K) indicate literature data (Kumagae et al., 1992); curves indicate correlation of the model.

**Figure 5:**
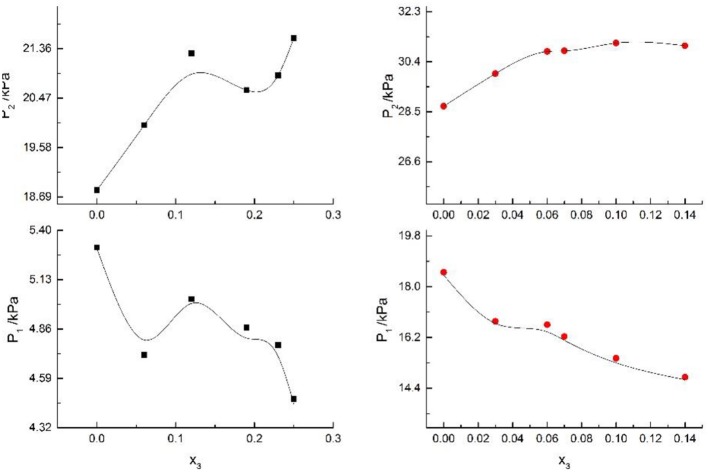
Correlation of VLE data of H2O(1)+ CH3OH(2)+NaCl(3) system. Filled symbols (■x2 = 0.45, T = 315 K; •x2 = 0.22, T = 335 K) indicate Literature data (Yang and Lee, 1998). Curves indicate correlation of the model.

**Figure 6:**
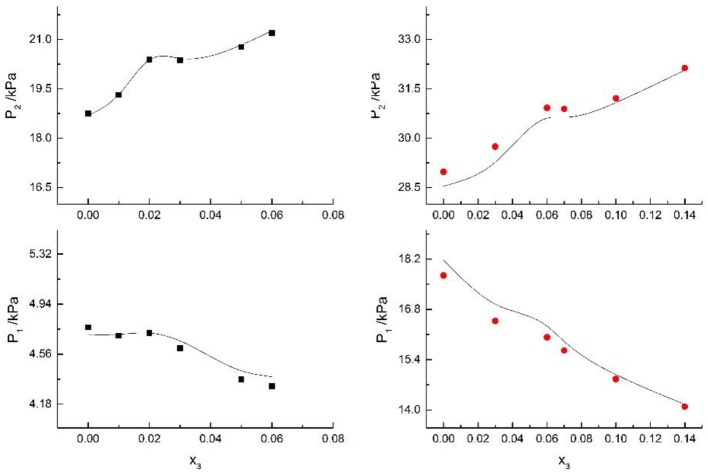
Correlation of VLE data of H2O(1)+ CH3OH(2)+NaBr(3) system. Filled symbols (■x2 = 0.45 and T = 315 K; •x2 = 0.22 and T = 335 K) indicate Literature data (Xu et al., 2018); curves indicate correlation of the model.

**Figure 7:**
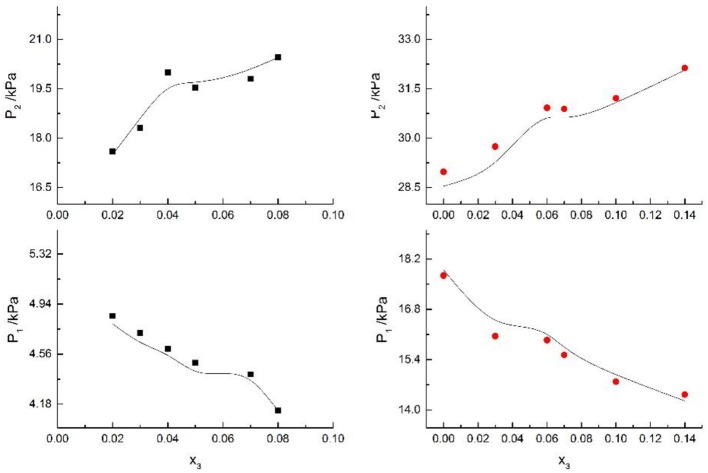
Correlation of VLE data of H2O(1)+ CH3OH(2)+NaI(3) system. Filled symbols (■x2 = 0.45 and T = 315 K; •x2 = 0.22 and T = 335 K) indicate experimental data; curves indicate correlation of the model.

**Figure 8:**
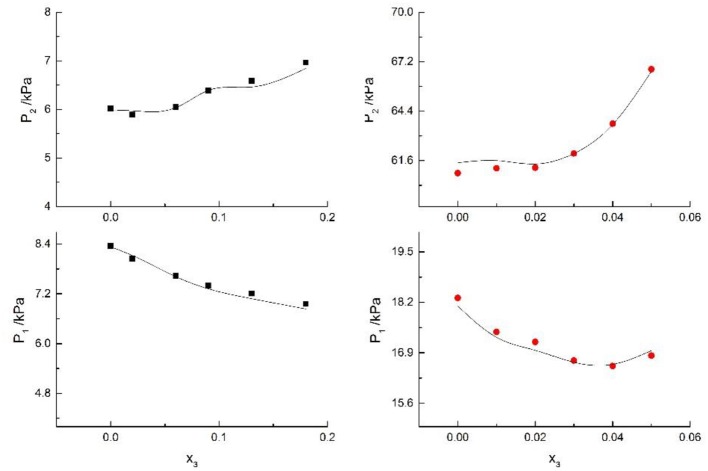
Correlation of VLE data for H2O(1)+ CH3OH(2)+KCl(3) system. Filled symbols (■x2 = 0.08 and T≈316 K; •x2 = 0.46 and T = 341 K) indicate Literature data (Xu et al., 2018); curves indicate correlation of the model.

**Figure 9:**
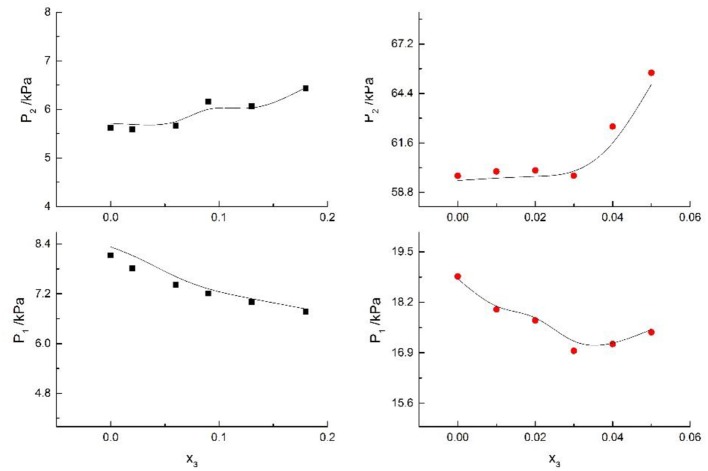
Correlation of VLE data for H2O(1)+ CH3OH(2)+KBr(3) system. Filled symbols (■x2 = 0.08 and T = 316 K; •x2 = 0.46 and T = 341 K) indicate experimental data; curves indicate correlation of the model.

**Figure 10:**
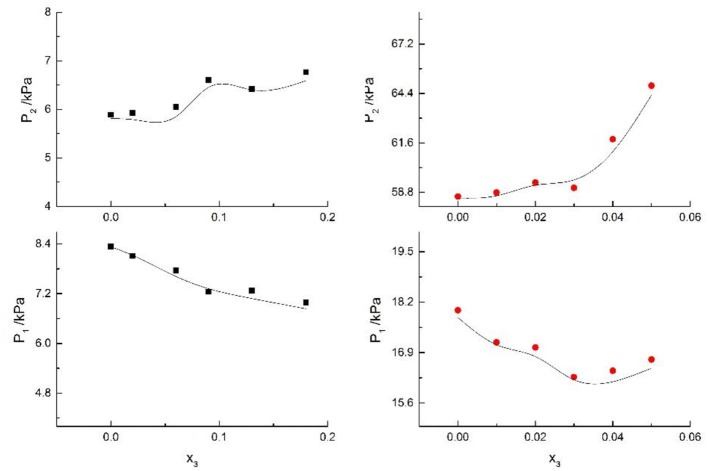
Correlation of VLE data for H2O(1)+ CH3OH(2)+KI(3) system. Filled symbols (■x2 = 0.08 and T = 316 K; •x2 = 0.46 and T = 341 K) indicate experimental data; curves indicate correlation of the model.

Seven salts (i.e., NaCl, NaBr, NaI, KCl, KBr, KI, and CaCl_2_) in water, methanol, ethanol, and normal propyl solvent systems were chosen to correlate the proposed new model, as shown in Table 5 and Figures 4–10. From the tables and Figures, small deviations can be found between literature data and calculated value, indicating a good accuracy of the proposed model for correlating the VLE behavior in mixed solvent electrolyte systems. The result indicates that model assumptions and derivations process are suitable for mixed solvent electrolyte systems.

### Comparison With Other Methods

We selected eight systems for comparing Yang's model (Yang and Lee, 1998), Iliuta's model (Kumagae et al., 1992), Kumagae's model (Robinson and Stokes, 2012), and Xu's model (Xu et al., 2018) with the proposed model in this work. Comparison results are shown in Tables 6, 7.

**Table 6:** Comparison of VLE for H_2_O-methanol-salt systems at 298.15 K.

**Systems**	**Data point**	**Pressure error dY/ kPa**			
		**Yang (Yang and Lee, 1998)**	**Iliuta (Iliuta et al., 2000)**	**Xu's model (Haynes, 2016)**	**This work**
H_2_O-Methanol-NaCl	20	0.42	0.20	0.083	0.03
H_2_O-Methanol-KCl	20	0.32	0.20	0.059	0.02
H_2_O- Methanol-NaBr	10	0.48		0.076	0.03
Mean value		0.41	0.20	0.073	0.027

*dY = (1/N)∑|P_exp_-P_cal_|, where N is the number of data points*.

**Table 7:** Comparison of VLE for systems containing CaCl_2_ at 298.15K.

**Systems**		**Kumagae (Kumagae et al.**, **1992****)**		**Xu's model (Xu et al.**, **2018****)**		**This work**	
	**Data point**	**dP_**p**_/%**	**dP_**x**_/%**	**dP_**p**_/%**	**dP_**x**_ /%**	**dP_**p**_/%**	**dP_**x**_ /%**
H_2_O+Methanol+CaCl_2_	40	6.12	1.38	6.47	1.64	3.79	1.08
H_2_O+Ethanol+CaCl_2_	20	2.61	0.40	1.77	1.57	2.32	0.30
Methanol+Ethanol+CaCl_2_	20	3.64	1.87	3.83	2.00	2.20	1.67
Methanol+1-propanol+CaCl2	36	3.69	1.23	3.24	1.99	3.42	1.07
Ethanol +1-propanol+CaCl2	36	2.14	0.82	2.1	0.9	3.14	1.01
Mean value		3.64	1.14	3.48	1.62	2.97	1.03

*dP_p_ = (1/N)∑|P_exp_-P_cal_|/P_exp_ × 100%, where N is the number of data points*.

*dP_x_ = (1/N) ∑|_exp_-x_cal_|/x_exp_ × 100%, where N is the number of data points*.

For water-methanol-salt systems (Table 6), the *dY* maximum value (*dY* = 0.03 kPa) of the proposed model in this work was less than that of Yang's model (*dY* = 0.42 kPa), Iliuta's model (*dY* = 0.2 kPa), and Xu's model (*dY* = 0.083 kPa). Likewise, the mean value *dY* (0.027 kPa) of the model in this work was less than that of Yang's model (*dY* = 0.41 kPa), Iliuta's model (*dY* = 0.2 kPa), and Xu's model (*dY* = 0.073 kPa). The specific assumptions and theoretical derivations for mixed solvent electrolyte systems were not introduced in Yang's model and Iliuta's model, which may have resulted in inaccurate model calculations for certain systems. The model in this work for the excess Gibbs energy was derived from the NRTL equation, and the activity coefficients were calculated by solvent-salt terms and solvent-solvent terms, respectively. In comparison with Yang's model and Iliuta's model, the assumption of solvation for mixed solvent electrolyte systems was introduced in this work. Due to the assumptions and theoretical derivations in this work, the proposed model in this work was considered to be more comprehensive and accurate.

For the systems containing CaCl_2_ in Table 7, the maximum value *dP*_*p*_ and *dP*_*x*_ of the proposed model were 3.79 and 1.67%, respectively. The maximum value *dP*_*p*_ and *dP*_*x*_ of Kumagae's model were 6.12 and 1.87%, respectively, and the maximum value *dP*_*p*_ and *dP*_*x*_ of Xu's model were 6.47 and 2.00%, respectively. The mean value *dP*_*p*_ and *dP*_*x*_ of the proposed model were 2.97 and 1.03%, respectively. The mean value *dP*_*p*_ and *dP*_*x*_ of Kumagae's model were 3.64 and 1.14%, respectively, and the mean value *dP*_*p*_ and *dP*_*x*_ of Xu's model were 3.48 and 1.62%, respectively. In this section, *dP*_*p*_ and *dP*_*x*_ were calculated via two equations:

(11) $$\begin{array}{l} dP_{P}=\left(1/N\right)\sum |P_{\exp}-P_{cal}|/P_{\exp}\times 100\% \end{array}$$

(12) $$\begin{array}{l} dP_{x}=\left(1/N\right)\sum |x_{\exp}-x_{cal}|/x_{\exp}\times 100\% \end{array}$$

From the results in Table 7, correlations of the proposed model in this work were better than Kumagae's model and Xu's model. Kumagae's model is a semi-empirical model based on Hála's model, and the model in this work simplified the calculation procedure as compared to Kumagae's model. In addition, Kumagae calculated the VLE of CaCl_2_+CH_3_OH+H_2_O and CaCl_2_+CH_3_CH_2_OH+H_2_O to be at 298.15K in the model. However, the developed model in this work was demonstrated as suitable for a broader range of temperature and pressure conditions. In summary, the proposed model was superior to other models in terms of the calculation results, calculation process, model comprehensibility, and scope of application.

## Conclusions

In this paper, the VLE data for H_2_O+CH_3_OH+NaI, H_2_O+CH_3_OH+KBr, and H2O+CH3OH+KI systems were reported. The reliability of measurements was verified by comparing our experimental data in two binary systems (i.e., H_2_O+CaCl_2_ and H_2_O+C_2_H_5_OH). Through the analysis, it has been shown that the solubility of salt is an important factor affecting the VLE.

Contemporaneously, a modified model was developed for calculating the VLE of mixed solvent electrolyte systems. The proposed model introduced a new excess Gibbs energy equation that is based on the NRTL model and Xu's model. We obtained the new model's parameters by correlating the experimental and literature data. The calculation results were compared to Yang's model, Iliuta's model, Kumagae's model, and Xu's model. In general, the model in this work can be used to successfully calculate VLE data for mixed solvent electrolyte systems.

## Data Availability Statement

All datasets generated for this study are included in the article/supplementary material.

## Author Contributions

XX and ZW: overall planning of the article and modeling. NZ: experimental design and data processing. YZ: experimental design and experimental equipment assembly. YW: experimental operation and data processing.

### Conflict of Interest

The authors declare that the research was conducted in the absence of any commercial or financial relationships that could be construed as a potential conflict of interest.

## Figures and Tables

**Table:** 

*a*	activity
*G^e^*	excess Gibbs energy, J•mol^–1^
*R*	gas constant, J•mol^–1^•kg^–1^
*m*	molality, mol•kg^–1^
*n*	mole, mol
*m_x_*	total molality of solute, mol•kg^–1^
*m_w_*	molar of free water, mol•kg^–1^
*h*	hydration numbers of the solute
Z	solvation parameters
*T*	temperature, K
*Ms*	molecular weight of water
γ	activity coefficients
*n_t_*	integral molar quantity, mol
τ	parameter
*i*	component *i*
*j*	component *j*
*dY*	meanabsolute error, mol•kg^–1^
*dP*	mean relative error, %
*dP_P_*	mean relative error for vapor pressure, %
*dP_y_*	mean relative error for vapor phase composition, %
*x_1_*	composition1 in liquid phase
*x_2_*	composition2 in liquid phase
*x_3_*	composition3 in liquid phase
*y_1_*	composition1 in gas phase
*y_2_*	composition2 in gas phase
